# Comparison of an Electrical Cranial Access Drill With Autostop Technology to a Traditional Hand Crank Cranial Access Drill

**DOI:** 10.1227/neuprac.0000000000000159

**Published:** 2025-08-22

**Authors:** John L. Kilgallon, Geoffrey R. O'Malley, Daniel Monahan, Shayan Sadegh, Harshal Shah, Ira M. Goldstein, Nitesh V. Patel

**Affiliations:** ‡ Department of Neurosurgery, Hackensack Meridian School of Medicine, Nutley, New Jersey, USA;; § Department of Neurosurgery, Hackensack University Medical Center, Hackensack, New Jersey, USA;; ‖ Department of Neurosurgery, HMH-Jersey Shore University Medical Center, Neptune, New Jersey, USA

**Keywords:** Burr hole craniostomy, External ventricular drain, Power drill

## Abstract

**BACKGROUND AND OBJECTIVES:**

Craniostomies performed at bedside are one of the most important procedures in neurosurgery allowing for cranial access for monitoring of intracranial pressure, evacuation of subdural or epidural hematomas, or the placement of external ventricular drains. Although neurosurgery as a whole has seen rapid advances in its technology, craniostomies continue to be performed with hand crank drill technology similar to what was used in the 1600s. The purpose of this study was to compare the efficacy and safety profile of a novel electrical cranial access drill with autostop technology (ECAD) to that of traditional hand crank drills.

**METHODS:**

Using both drills, holes were drilled into the cranial vault of human cadavers by a veteran cranial surgeon and by a medical student without prior experience in the procedure. Time to drill each hole and the number of dural violations was compared between drills.

**RESULTS:**

Overall, 30 craniostomies were created with the hand crank drill and 61 were created with the ECAD. The average time to hole competition was significantly longer with the hand crank drill than with the ECAD (24.1 vs 16.5 seconds, *P* < .001). There were significantly more dural violations with the hand crank drill than with the ECAD (13 vs 2, *P* = .002), which engaged autostop in 100% of procedures.

**CONCLUSION:**

The electric drill with autostop technology demonstrated faster time to hole completion and significantly fewer dural violations than the traditional hand crank drill.

**ABBREVIATIONS:**
ECADs: electrical cranial access drills with autostop technology
NNT: Number Needed to Treat
OR: operating room.

The evolution of cranial access techniques has been pivotal in advancing neurosurgical care of patients with intracranial pathology. Hand crank craniostomies, traditionally known as twist drill craniostomies, remain a critical surgical technique in modern neurosurgery, especially for emergency interventions where rapid decompression is needed.^1^ These procedures are typically performed using a manual drill to create one or more small holes in the skull. The primary indications for this method include the evacuation of subdural hematomas, chronic subdural hematomas, and intracranial pressure (ICP) monitoring. The introduction of electrical drills represents a further step toward increased efficiency in performing these procedures.^2-5^ Similarly, an important consideration in implementing these tools is the minimization of risks associated with cranial access, with the goal of reducing the incidence of inadvertent dural or cerebral injury during drilling.

Another consideration is the setting in which these tools are used. Cranial access procedures are commonly performed in both the intensive care unit (ICU) and the operating room (OR), depending on the urgency and complexity of the case. In the ICU, quick access to the cranial cavity is often required for emergency interventions such as the management of acute ICP. By contrast, the OR allows for more elaborate procedures that may involve complex dissections and careful manipulation of brain tissue. In very acute patients, these procedures are also performed in the emergency department

Despite advancements, however, current cranial access methods in both the ICU and OR face significant challenges. In the ICU, the urgency of procedures often necessitates rapid cranial access, which can increase the risk of complications such as hemorrhage or infection. Similarly, in the OR, even with controlled conditions, the precision required for safe cranial access can be compromised by the limitations of manual tools, which may not always provide the necessary control or feedback to prevent tissue damage.^2,4^ Currently, in ICU or at the bedside, the hand crank is commonly used. Alternatively, in the OR, drills are often electrical and an autostop perforator is available. However, the autostop drill in the OR is a larger diameter than what is necessary for bedside cranial access for external ventricular drain or ICP monitor placement; thus, specialized electrical cranial access drills with autostop technology (ECADs) tailored to use in the ICU and at the bedside have been developed to bridge these gaps.^4^ This type of tool could also improve access to proper neurosurgical care in areas where non-neurosurgeons may be tasked with performing craniostomies.

The primary goal of this study, therefore, is to evaluate the efficacy and safety of a novel ECAD compared with traditional hand crank drills, specifically based on the time required to gain cranial access and the rates of dural violation for each method. This new technology promises to enhance surgical precision by automatically stopping the drill on breach of the skull before puncturing the dura, reducing the risk of damage to brain structures; while dural violation is inevitable in gaining intradural access, ECAD can help avoid it occurring in an uncontrolled fashion. By systematically comparing these two methods, this study aims to provide evidence-based recommendations that could lead to widespread adoption of safer, more effective cranial access techniques.

## METHODS

Two different operators conducted the drilling procedures. Operator 1 was a fourth year medical student with no previous experience in performing craniostomies on training devices, cadavers, or patients. Operator 2 had 10 years of neurosurgery experience. Two formalin preserved cadaveric skull vertices with intact dura were used in the study. No brain was involved in the specimens used, and therefore, the depth of each plunge associated with the dural violations could not be measured. Standard ethical protocol for cadaveric studies was followed regarding the handling of the skull cap. Institutional Review Board approval and patient consent to procedure were not required as no patient data or interactions were involved in this study.

**FIGURE 1.**
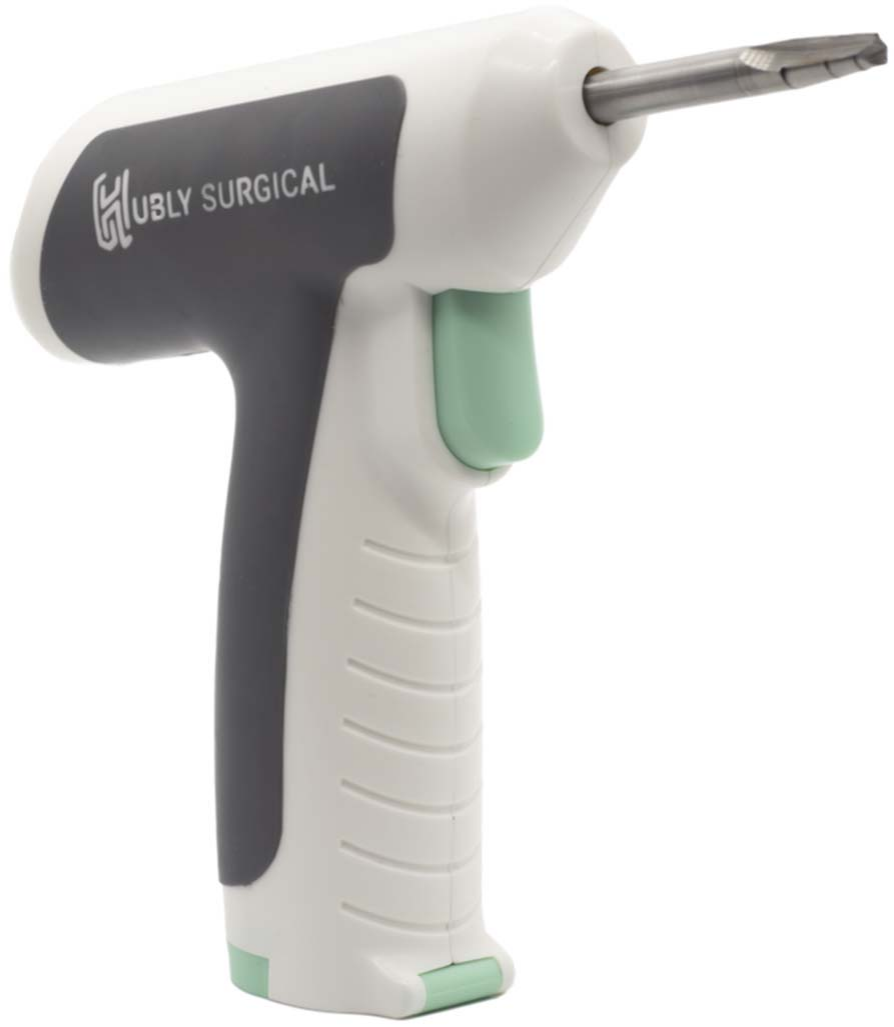
Image of the electrical cranial access drill with autostop produced by Hubly (Hubly Inc.).

**FIGURE 2.**
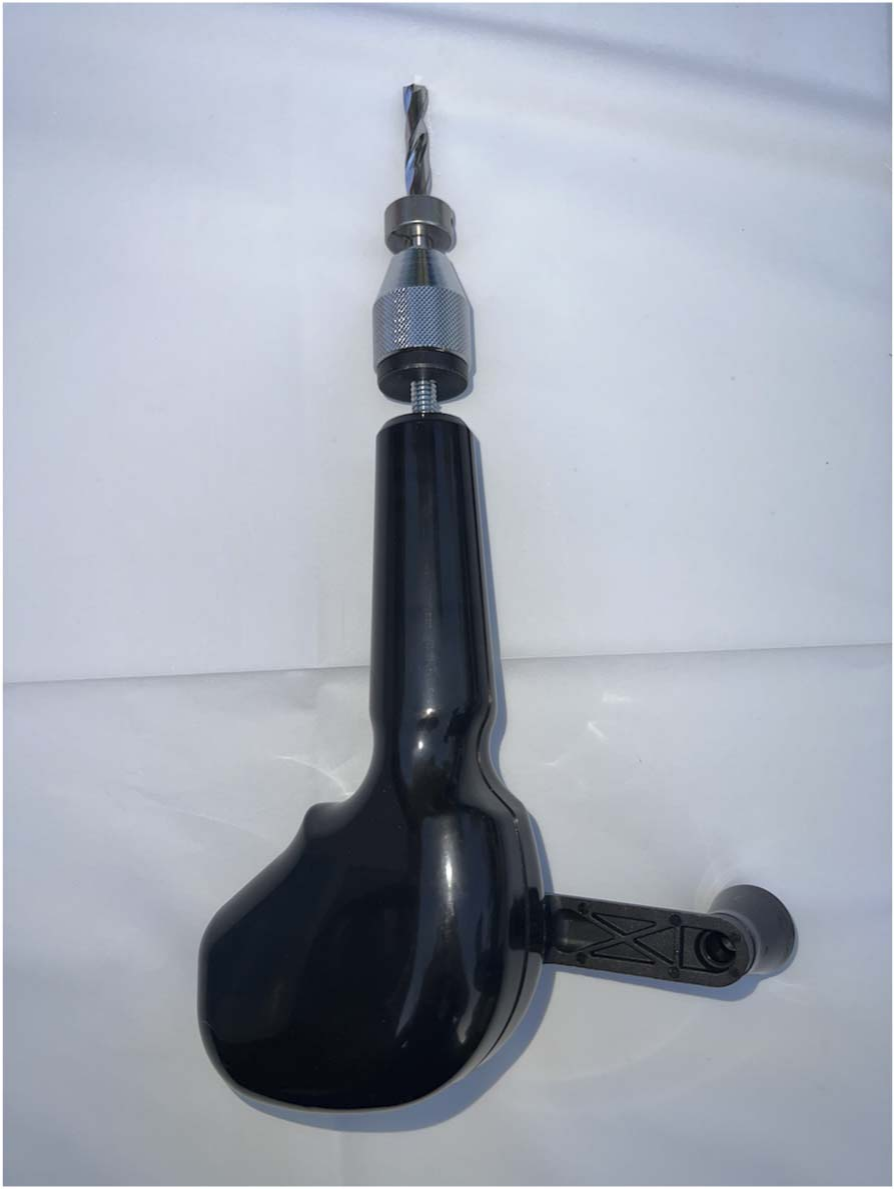
Image of the hand crank drill produced by Integra (Integra LifeSciences).

**FIGURE 3.**
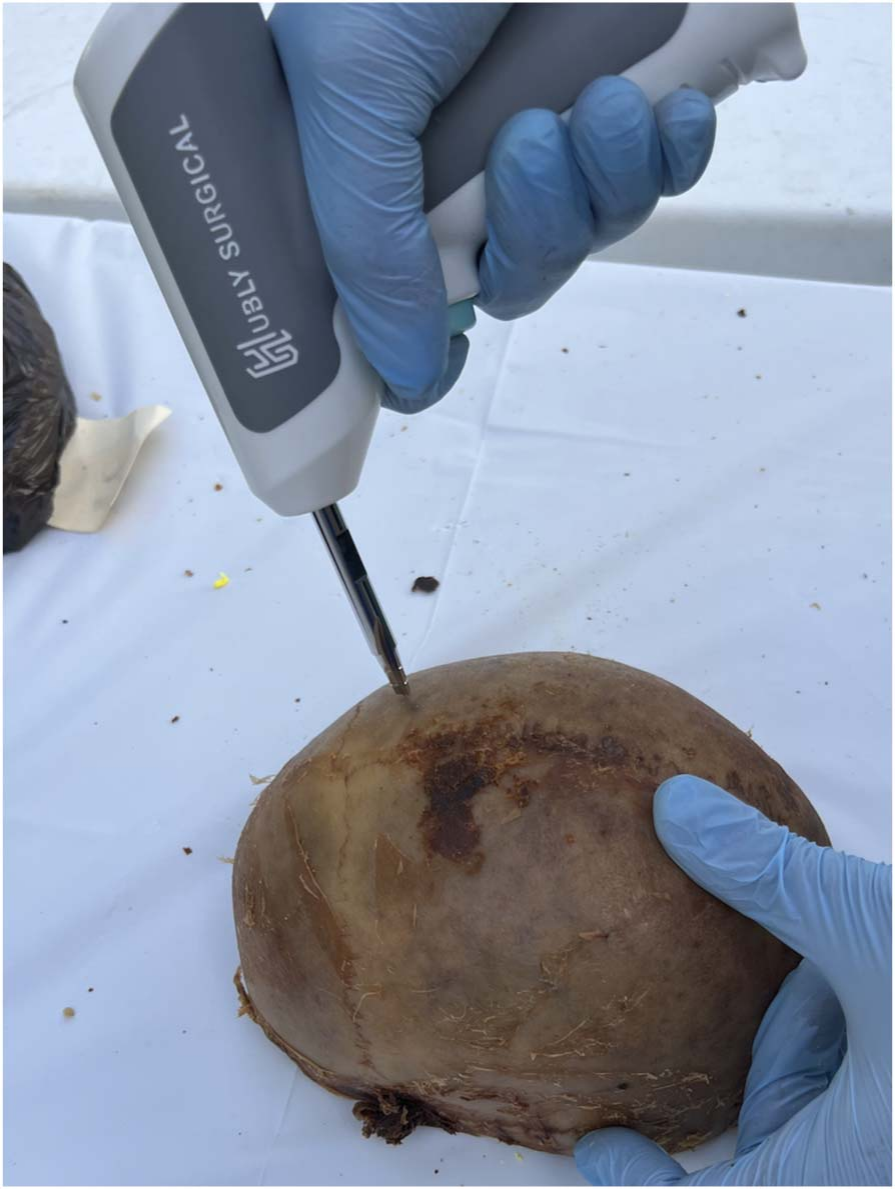
The experimental setup is demonstrated with the electrical cranial access drill.

**FIGURE 4.**
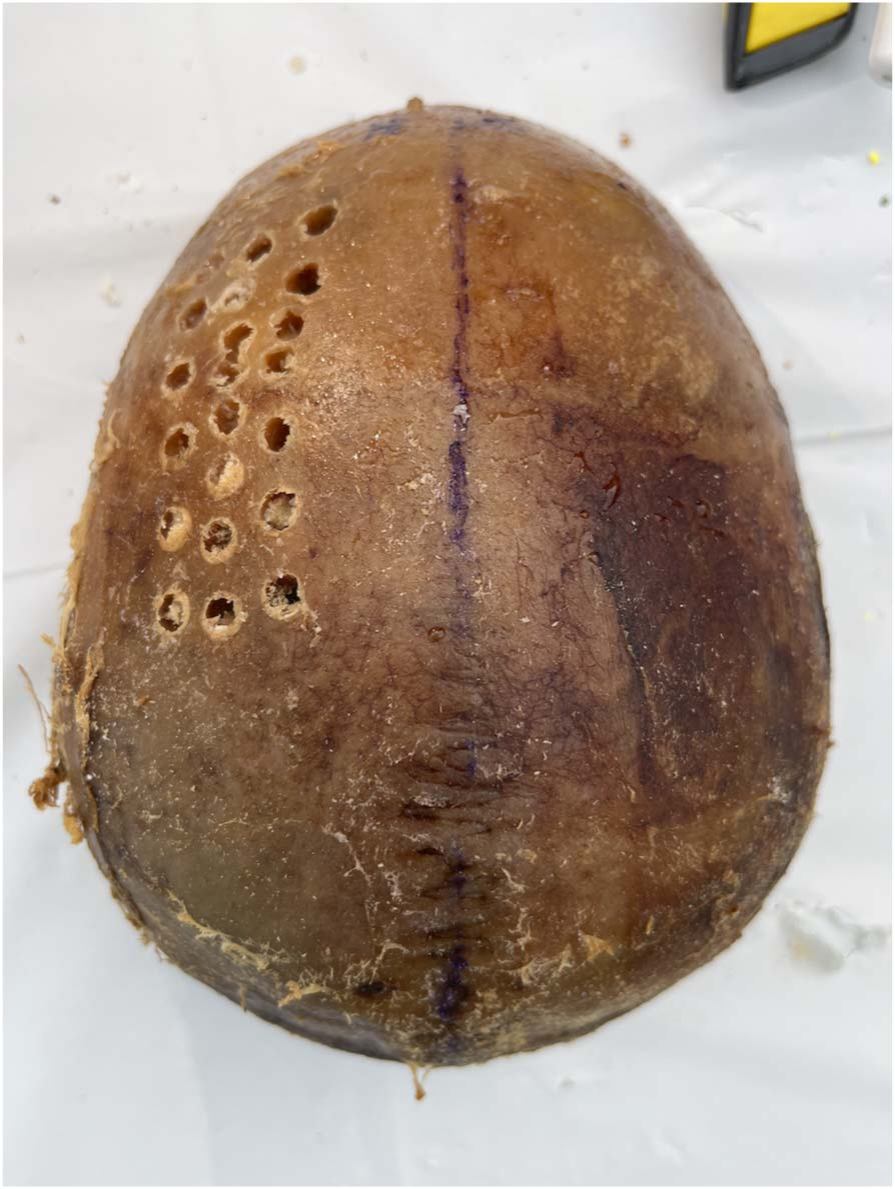
Appearance of the craniostomies on the skull cap.

In a laboratory setting, each skull cap was placed on a table and secured by the hands of the nondrilling operator to simulate a bedside procedure. Two different types of devices were used by each operator: a novel ECAD (Figure 1) and a traditional hand crank drill with the positive stop thumb screw set at a depth of 2 cm (Figure 2). The Hubly Drill (Hubly Inc.) is a device approved by the Food and Drug Administration (FDA) to use in a clinical setting with a 510(k) number of K230619. Each operator's drill attempts with the ECAD and hand crank drill were performed on opposite sides of the same skull cap. The experimental setup is presented in Figure 3 with the ECAD. Figure 4 shows the appearance of the craniostomies on the skull caps.

The number of attempts was recorded for each type of drill. For each attempt, variables collected included the time to drill (seconds), whether there was a dural violation based on visual inspection, the location of the hole (frontal-parietal or temporal), and the number of times the drill bit had been used.

All statistical analyses were performed using IBM SPSS Statistics, version 29.0 (IBM). The Shapiro-Wilk test was used to analyze the normality of the distribution of the time to hole completion and dural penetrations. When both distributions were determined to be nonparametric, the Mann-Whitney *U* test was performed to compare between the ECAD and a traditional hand crank drill.

## RESULTS

Overall, there were 15 hand crank drilling attempts each for operator 1 and operator 2 (30 total); there were 31 electric drilling attempts for the medical student and 30 for the attending surgeon (61 total) (Tables 1 and 2). The average time to drill was significantly longer using the hand crank drill at 24.1 seconds (SD 9.3) as compared with 16.5 seconds (SD 10.0) with the electric drill (95% CI [3.68, 11.50]; *P* < .001). Similarly, there were a significantly greater number of dural violations for the hand crank drill compared with the electric drill, with 13 (43.3%) vs 2 (3.3%), respectively (risk difference 95% CI [−58.2%, −21.9%]; *P* = .002).

**TABLE 1.** Dural Violations by Attempt by Operator With the Hand Crank Drill

Attempt number	Operator 1		Operator 2	
	Dural violation?	Drill bit use number	Dural violation?	Drill bit use number
1	No	1	No	2
2	No	2	No	3
3	No	3	No	4
4	No	4	No	5
5	Yes	5	Yes	6
6	Yes	6	Yes	7
7	Yes	7	Yes	8
8	No	8	No	9
9	Yes	9	Yes	10
10	Yes	10	Yes	11
11	No	11	No	12
12	Yes	12	Yes	13
13	Yes	13	Yes	14
14	No	14	No	15
15	Yes	15	Yes	16
Total dural violations	5		8	

**TABLE 2.** Dural Violations by Attempt by Operator With the electrical cranial access drill With Autostop Technology

Attempt #	Operator 1		Operator 2	
	Dural violation?	Drill bit use number	Dural violation?	Drill bit use number
1	No	1	No	6
2	Yes	2	No	7
3	No	3	No	8
4	No	4	No	9
5	No	5	No	10
6	No	6	No	11
7	No	7	No	12
8	No	8	No	13
9	No	9	No	14
10	No	10	No	15
11	No	11	No	16
12	No	12	No	17
13	No	13	No	18
14	No	14	No	1
15	No	15	No	2
16	No	16	No	3
17	No	17	No	4
18	No	18	No	5
19	No	19	Yes	6
20	No	20	No	7
21	No	21	No	8
22	No	22	No	9
23	No	23	No	10
24	No	24	No	11
25	No	25	No	12
26	No	26	No	13
27	No	27	No	14
28	No	28	No	15
29	No	29	No	16
30	No	30	No	17
31	No	31	-	-
Total dural violations	1		1	

For operator 1, using the electric drill resulted in a mean time to drill of 12.66 seconds and 1 dural violation, while the traditional hand crank drill included a mean time to drill of 16.87 seconds and 5 dural violations. On the other hand, operator 2 using the electric drill resulted in a mean time to drill of 20.82 seconds with 1 dural violation, while the traditional hand crank drill included a mean time to drill of 31.37 seconds with 8 dural violations. For interoperator variability, operator 1 had a significantly lower mean time to drill for both the electric drill and hand crank drill (95% CI [3.6-12.8 seconds] and [11.3-17.7 seconds], respectively; *P* < .001), and there were no statistically significant differences in dural violations.

## DISCUSSION

In comparing the efficacy of a novel ECAD to that of a traditional hand crank drill, we found that the electric drill was both more efficient and safer across multiple levels of surgical expertise. These results are likely due to the fact that the electric drill allows for faster drilling, while the autostop technology protects against dural violation after perforation of the skull. In addition, these results were consistent between operators, indicating that the electric drill has similar benefits in operators with differing levels of experience. Interestingly, the less experienced operator performed better in both efficiency and dural preservation than the more experienced operator. However, this could be due to the fact that different cadaver skulls were used or that it was the first time either operator had used the drill in this setting.

Recent studies have evaluated the effectiveness and safety of modern cranial access technologies, specifically comparing the autostop cranial access technology with traditional hand crank drills. The autostop technology incorporates a smart autostop mechanism that automatically disengages the drill to prevent dural penetration, enhancing safety during craniotomies. This technology has been shown to engage autostop in 100% of procedures, effectively preventing dural penetration in 99.4% of cases.^2^ By contrast, traditional hand crank drills lack an autostop mechanism, requiring manual control to avoid damaging the dura.^6^ These drills are typically used in emergency situations due to their simplicity and portability. However, they demand high precision and caution from the operator to prevent neurological complications associated with unintentional dural penetration.^2^

An additional consideration when comparing the two drilling methods is the learning curve involved for each. One potential benefit of electric drills such as the one used in this study is that they are designed with safety mechanisms that automatically prevent the drill from penetrating too deeply, which can reduce the risk of accidental neurological injuries. This may also contribute to a shorter learning curve due to their automated features. By contrast, hand crank drills require a higher degree of manual skill and experience. The lack of an autostop feature means that surgeons must rely heavily on their technique and tactile feedback to avoid damaging critical structures. This may lead to a steeper learning curve, as practitioners must develop a finely tuned sense of depth and pressure to use these tools safely and effectively.^7^ A less obvious but still relevant component of the learning curve is drill setup time. Depending on the manufacturer of the hand crank drill, the bit may come preattached or need to be placed into the drill itself. Placement time, although greatly varies, along with potential need for readjustment of tightness, must be considered. The novel ECAD device comes as an all-in-one system with the bit already built into the construct, making it immediately deployment ready.

Dural overpenetration, or “plunge,” is a concerning complication of dural access procedures. Penetration into the dura can result in a range of complications such as intracranial hemorrhage, seizures, and death.^8^ Reported rates of dural tear during electric drill craniostomies without autostop technology by experienced neurosurgeons have previously ranged from 20% to 30%,^9^ while on the rates of dural plunge events from hand-crank craniostomies is limited. Traditional hand-crank drills aim to avoid plunge events by relying on depth-controlling cuffs, in conjunction with measured bone width from computed tomography scans. By contrast, some electrical drills have aimed to prevent plunge events through various mechanisms, including manual release on a drop in torque visualized on a monitor, an automatic stop based on a change in thrust force and torque, and algorithms detecting changes in bone layers that intentionally allow a thin layer of bone to remain. The electric drill instead relies on proprietary software and hardware, as well as a tapered drill bit that provides secondary protection. However, it is important to note that risk of dural laceration still exists with the use of automatic-releasing cranial perforators.^10^ Furthermore, the shelf life of the electric drill is a notable factor; as a battery-powered device to be used in emergency situations, it would potentially be a fatal problem if there is a mechanical issue or if a dead battery is encountered.

Finally, in the evaluation of the electric drill, a significant metric derived from our study is the Number Needed to Treat (NNT) to prevent an additional dural plunge event. The analysis reveals that with an Absolute Risk Reduction of 40% (calculated from a control event rate of 43.3% with the hand crank drill and an experimental event rate of 3.3% with the electric drill), the NNT is approximately 2.5. This indicates that for every 2 to 3 patients treated with the electric drill instead of the traditional hand crank drill, one additional dural plunge event is prevented. However, this figure does not account for the resultant costs of increased length of stay, increased number of scans, or increased likelihood of readmission. This calculation underscores the efficacy of the electric drill in enhancing safety during cranial access procedures, significantly reducing the risk of dural violation which can lead to severe neurological complications.

### Limitations

One limitation to this study, as mentioned in prior research on the topic, is that dura mater in cadavers tends to be more rigid compared with that in live patients, which may lead to an underestimation of dural penetration by the drills tested^2^; similarly, it is also possible that cadaveric dura is more brittle and less flexible, which could increase the likelihood of a dural puncture. In addition, the study did not account for variations in skull density or thickness, which can affect the performance and safety of the drills, and while the study provides quantitative data on dural violations and drilling time, it does not extensively address other potential complications such as infection or hemorrhage that might arise from the use of these drills in a clinical setting. Furthermore, the fact that only 2 operators were included in the study limits the generalizability of the findings, as individual skill levels and experiences can influence outcomes; this suggests that larger-scale studies with multiple operators possessing varying levels of experience are necessary to fully characterize the performance benefits and limitations of ECAD compared with traditional twist drills. In addition, although our analysis identified an NNT of approximately 2.5 to prevent one dural plunge event using the ECAD compared with the hand crank drill, the clinical significance of this finding warrants careful interpretation. Dural plunges, while potentially serious, do not universally lead to clinically meaningful adverse outcomes, particularly because bedside craniostomies are frequently performed at Kocher point—a location traditionally chosen due to its lower risk of significant neurological injury in the event of inadvertent dural penetration. Finally, while the results demonstrated statistically significant improvements in drilling efficiency using the ECAD, with an average reduction of approximately 8 seconds compared with the traditional hand crank drill, the clinical significance of this specific time saving remains unclear. The clinical effect of this specific magnitude of time savings is uncertain and warrants further investigation through clinical studies assessing real-world patient outcomes. Future studies should focus on evaluating whether the observed reduction in craniostomy completion time translates into clinically meaningful differences in patient morbidity, mortality, or other pertinent clinical parameters.

## CONCLUSION

This study systematically evaluated the efficacy and safety of a novel ECAD compared with traditional hand crank drills in neurosurgical settings. The findings revealed that the electric drill was more efficient and safer, significantly reducing the time to drill and the incidence of dural violations, thereby enhancing surgical outcomes. However, limitations include the use of cadaveric models, which might not accurately represent live tissue responses, as well as the limited number of operators, which could affect the generalizability of the results. Future studies should explore the performance of these drills in live clinical settings and consider a broader range of operators to validate and extend these findings. In addition, further research could investigate the long-term outcomes of patients undergoing procedures with these technologies to better understand their impact on patient recovery and complications.
